# Choosing Sustainability: Decision Making and Sustainable Practice Adoption with Examples from U.S. Great Plains Cattle Grazing Systems

**DOI:** 10.3390/ani12030286

**Published:** 2022-01-24

**Authors:** Amber Campbell, Audrey E. H. King

**Affiliations:** 1 National Institute of Food and Agriculture, USDA, Washington, DC 20250, USA; amber.campbell@usda.gov; 2 Rural Renewal Initiative, Oklahoma State University, Stillwater, OK 74078, USA

**Keywords:** behavior change, practice adoption, farmer decision making, grazing, sustainability

## Abstract

**Simple Summary:**

Sustainable intensification of beef cattle production systems will involve widespread adoption of new practices and technologies. Whether these techniques involve genetic enhancement of cattle or forages, new technologies, or a combination of existing management practices, behavioral change on the part of beef cattle producers will be required. Many factors contribute to the likelihood of these changes. Concepts from the social science of behavior change will be useful to scientists and Extension and other professionals wanting to encourage the adoption of new practices and technologies related to sustainable intensification of beef cattle production. This will include consideration of the context of the farms on which they would be used and the farmers who they hope to adopt them, as well as how the practices will be perceived by those farmers. These will influence producer beliefs and attitudes about the natural and social consequences of adoption in addition to its possibility. Integrating these concepts throughout the development and promotion of new practices and technologies may increase the likelihood of widespread adoption.

**Abstract:**

Sustainable intensification of animal agriculture will rely on the acceptance and adoption of many new practices and technologies. We discuss the literature related to behavior change and sustainable practice adoption in the context of beef cattle production, focusing on sustainable rotational grazing and the use of cover crops. Research from a variety of contexts is discussed with a conceptual framework that combines diffusion of innovation theory with the reasoned action approach. Background characteristics of producers and their operations as well the characteristics of any new practice/technology will influence producer perceptions of them. These background and perceived practice characteristics will influence producer behavioral, normative, and control beliefs regarding the behavior, which will in turn inform attitudes about the behavior and perceptions regarding behavioral norms and the capacity to adopt new behaviors. Factors such as the demographics of beef cattle producers, land tenure, and labor and credit availability, as well as producers’ concepts of what it means to be a “good farmer”, should inform the conceptualization and development of new practices and technologies to increase the likelihood of their adoption.

## 1. Introduction

Grassland pasture and range is the largest land use type in the United States, with over 655 million acres (29% of U.S. agricultural land) [1]. Well-managed grazing lands provide a wide array of ecosystem services, including soil carbon sequestration, reduced erosion, water quality and storage improvements, wildlife habitat and recreational areas, and biodiversity [2,3,4,5]. Temperate grasslands are also among the most endangered ecosystems [6]. DeLonge and Basche [2] suggested the identification of best management practices (BMPs) that both maximize ecosystem services and maintain farm profitability as a strategy to provide economically and environmentally sustainable protection to these endangered ecosystems. Given the limited availability of additional land that can be utilized without deforestation, increased productivity of grazing-based systems both per animal and per unit of land is needed [7]. Sustainably intensifying grazing-based animal production systems will be essential to protect and preserve these ecosystems while also providing for the protein needs of a growing population. Approaches to the sustainable intensification of livestock production on pastures include the diversification of plants and ruminant species, improvement in feeding techniques and grazing management, plant breeding for improved nutrient use efficiency, integrated crop–livestock systems (ICLSs), and silvopasture systems [8]. Some have made a distinction between sustainable intensification and agroecology based on their methods and aims [9]. Sustainable intensification focuses on the use of technologies to increase efficiency on existing agricultural lands to prevent further encroachment on remaining natural ecosystems. Agroecology applies ecological and social principles to agricultural systems [10] to reduce dependence on chemical inputs and production costs [11]. Sustainable intensification and agroecological practices are not mutually exclusive, and the considerations around practice adoption apply to all practices. Lampkin et al. [12] suggested that “agroecology can be seen as part of a broader approach to sustainable intensification focusing on ecological (or eco-functional) and knowledge intensification alongside technological intensification”.

An example of sustainable intensification in grazing systems includes management-intensive rotational grazing systems. In these systems, short-term grazing periods and longer rest periods are used to increase the carrying capacity of grazing lands [13]. An agroecological approach to grazing lands that incorporates management practices can effectively manage resources to improve soil health, sequester carbon, and prevent environmental damage [14]. Agroecological systems such as ICLS, including cover crops and no-till practices, support self-regulation within systems and enhance resilience while increasing productivity and ecosystem service provision [15]. BMPs represent practical ways to conserve soil, reduce water pollutants, and improve the productivity of agricultural lands [16,17,18].

Innovations toward sustainable intensification have been made in the beef industry in terms of pasture management, cattle selection tools to improve feed efficiency, and improvements in reproductive efficiency [19]. Moreland and Hyland [19] noted the need for improved communication between developers and end users of innovations in beef cattle production. Although many new technologies and practices are being developed, many of the practices that can be used to make animal agriculture more sustainable have been well known for decades. Despite long histories of promotion, many groups have expressed frustration at the low adoption levels of best management practices such as crop–livestock integration, cover crop grazing, rotational grazing, etc. [20]. Gillespie et al. [21] referred to beef cattle producers who are aware of more sustainable or productive practices but choose not to implement them in his or her operation as an “unexplained phenomenon”. They are not alone. The authors’ personal experiences with Extension professionals include laments such as, “We’ve been telling them to do the same thing for 30 years and they just won’t do it”. The frustration expressed is based on the assumption that sufficient information about a practice should ensure its adoption. This information deficit model assumes that a lack of knowledge or understanding of a practice is the only constraint to its adoption and that additional information will inevitably lead to changes in practice [22]. However, this ignores other, often significant, barriers and constraints to practice adoption.

Knowledge of the existence of a practice and some basic information on implementation are obviously necessary before a producer can possibly adopt a new practice, but as any Extension practitioner can attest, knowledge is far from sufficient to ensure adoption. Behavior change decisions, like the adoption of a new practice or technology, are complex and are influenced by past experiences, individual perspectives, motivations, and constraints [20]. Whether or not producers adopt practices that will enable them to sustainably intensify production in their operations is influenced by a number of factors, including their perceptions of new practices and whether they see them as relevant or feasible.

### Behavior Change Models

There is significant variation in the production practices and management decisions made by the more than 700,000 beef cattle producers in the U.S. alone [23]. A number of models have been developed to better understand behavioral change. These models are often used in research related to agricultural practice adoption. We discuss two of the primary models used to explain behavior change and practice adoption: perceived practice characteristics from Diffusion of Innovation (DOI) theory and Reasoned Action Approach (RAA).

DOI theory [24] focuses on how new technologies or practices spread within a social system. It identifies five categories of adopters: (1) innovators, (2) early adopters, (3) early majority, (4) late majority, and (5) laggards. These categories have varying levels of interest and desire to change their behavior. Five categories of perceived practice characteristics that influence practice adoption decisions are also identified: (1) relative advantage over the current practice, (2) compatibility, or the alignment of the practice with the values, experience, or perceived needs of the farmer, (3) perceived complexity or difficulty of adopting the practice, (4) trialability of the practice prior to large-scale adoption, and (5) observability of the results achieved by others who already adopted a practice. Thus, DOI theory explains the adoption of a new practice as resulting from a combination of the inherent characteristics of the potential adopters, independent of the innovation and their perceptions of the innovation. Dissemination models used to encourage innovation adoption go beyond passive DOI and actively providing information about innovative practices via change agents or intermediaries, often governmental officials [19].

The RAA [25,26] focuses on attitudes, behavioral norms, and perceptions of one’s efficacy to explain behavior change. It identifies three types of beliefs about a practice that influence adoption: (1) behavioral beliefs that focus on the consequences of practicing a behavior, (2) normative beliefs about the social acceptability of the behavior, and (3) control beliefs about the feasibility of a behavior [26]. According to RAA, attitudes toward a behavior or practice are the result of readily accessible beliefs and happen automatically as a result of the beliefs held about its attributes [27]. These are incorporated with other background factors to explain decisions to change behavior (i.e., adopt a practice) [25].

**Figure 1:**
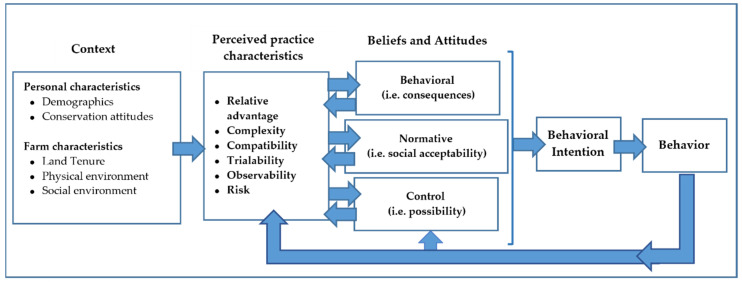
Conceptual model, adapted from Reimer [28] and Arbuckle and Roesch-McNally [29].

Reimer et al. [28] incorporated perceived practice characteristics from DOI theory [24] into the RAA model to describe the beliefs held about the attributes of a practice. They added riskiness as an additional perceived practice characteristic. This combined model was later used by Arbuckle and Roesch-McNally [29] to study cover crop adoption among Iowa farmers. We have adapted this model to acknowledge the interaction between perceived practice characteristics and behavioral, normative, and control beliefs. This model incorporating contextual factors, perceived practice characteristics, and producer beliefs about the natural and social consequences of a behavior, as well as their beliefs about their own ability, can be utilized to better understand the potential for behavior change. Consideration of these concepts can assist researchers in understanding producer behavior, developing sustainable practices and technologies to intensify beef cattle production that are more acceptable, and promoting their adoption. Figure 1 highlights the key aspects of the model.

## 2. Context Is Crucial

The decision to adopt or not adopt a practice begins with a producer’s perception of the practice. These perceptions and the expectations that producers create about a practice are informed by “the process of learning and experience, the characteristics and circumstances of the landholder within their social environment and the characteristics of the practice” [20]. While the characteristics of the practice itself are meaningful, how those characteristics are interpreted is dependent on what the producer already knows or has experienced. Other contextual factors, for instance, where and how the practice will be used, are also important to consider.

### 2.1. Farmer Characteristics

Often, characteristics of producers and their farms, rather than characteristics of the practice, have a strong influence on practice adoption. A substantial body of research examines the many characteristics of practice adopters. The size of operations, farm income dependence, and employment status are characteristics that are related to the use of BMPs in Oklahoma [30]. Producers who were dependent upon cattle for income were 10.2% more likely to know how to set optimal stocking rates. Moreover, if a producer worked off-farm, they were also more likely to set proper stocking rates. Age, which is addressed in the next section in greater detail, was a negative predictor of adoption in this study [30]. The covariance of age and off-farm work (i.e., retirement from other employment) was not addressed. In the same study, producers who used wheat as a forage viewed stocking rates to be critical and were likely to stock at lower rates in order to ensure adequate forage availability. A study examining cow-calf producers in Oklahoma found that dependence upon income from cattle and higher levels of education increased the likelihood that BMPs would be adopted, whereas the higher the age of a producer, the less likely they were to have adopted BMPs [31].

#### 2.1.1. Demographics and Labor Availability

The average age of a farmer in America has been increasing for decades [32]. This is especially true in animal agriculture. Respondents to a 2016 survey of cattle producers in the Southern Plains had an average age of 67 [33], a full decade older than the average age of all producers (57.5 years) according to the 2017 Census of Agriculture [32]. In many cases, producers are nearing retirement or mortality without a succession plan in place [34]. This disincentivizes the adoption of many new practices that require substantial costs to implement and become profitable only over time. In addition, the average age of producers may reduce the ability to implement practices that require additional manual labor. Insufficient access to labor, especially skilled labor, has been noted as a constraint on the adoption of new practices in the U.S. [33]. Among Australian beef producers, labor shortage was noted as a barrier to the adoption of ICLS [35]. A lack of access to skilled labor can also influence perceptions of the complexity of a practice and beliefs about their ability to adopt it [36]. These demographic and labor shortage concerns suggest that it may be beneficial to focus on the development of practices and technologies that have shorter intervals to profitability and reduce labor requirements over those that have extended profitability horizons or that increase labor requirements.

#### 2.1.2. Conservation Attitudes

Farmers and ranchers consistently self-identify as land stewards, and their identity as a *steward of the land* is a key aspect of what it means to be a *good farmer* [37,38]. These conservation attitudes have been studied as motivators for adopting more sustainable or conservation practices in a variety of contexts. Conservation attitudes provided intrinsic motivation for the adoption of conservation BMPs among Australian graziers [39]. A meta-analysis of conservation practice adoption found a positive association between adoption and farmer self-identification as primarily stewardship motivated or environmentally minded, rather than financially motivated [40]. Ryan, Erickson, and De Young [41] found that intrinsic motivations were the strongest motivators toward adopting BMPs. These intrinsic motivators included feeling connected to the land and a desire to maintain fruitful land for future generations. In the same study, economic compensation was the lowest-rated motivation category. Floress et al. [42] suggested reconsideration of focusing on the economic incentives for the adoption of conservation practices given the importance of stewardship attitudes in adoption decisions. However, among respondents to a survey of the California Cattlemen’s Association, the majority (68%) responded that they would prioritize economic viability if environmental protection and economic viability were in direct conflict [43]. 

### 2.2. Farm Characteristics and Context

The characteristics and attitudes of producers are not the only factors to consider in promoting new practices and technologies. If one would like to promote the adoption of more sustainable practices, it is important to first understand the context in which the practices are being adopted [44].

#### 2.2.1. Land Ownership and Tenure

Land ownership status is a key determinant of whether or not some practices are adopted. In some cases, management decisions such as whether or not a new practice can be implemented are determined by the landowner but may also be left up to the leaseholder. Absentee landlords are disconnected from the industry and do not necessarily understand the needed improvements or the financial burdens that producers face. Furthermore, the length or permanency of land lease agreements is also a factor in practice adoption. Producers are hesitant to pay for improvements, such as additional fencing and removal of brush and trees, or even to rest overused areas [44].

Producers who own the land that they graze have more freedom to implement practices without interference or the need to seek approval by landowners. They also have a greater incentive to adopt practices with a significant delay between initial implementation and economic return. King’s [44] previous work in Oklahoma identified the leasing structure imposed on Oklahoma school land as a major barrier to the adoption of practices, with a return horizon beyond five years. This system gives no preference to the former lessee in a five-year bid lease system. While the system optimizes revenues benefitting state schools, those leasing the land could not justify making improvements to the land that would require more than five years to recoup the initial costs. Not only were producers making improvements unable to ensure they would have access to the land for more than five years, but the price bid by others and the cost of retaining the land would likely increase as a result of the improvements [45].

#### 2.2.2. Physical and Social Environment

Producers’ physical and social environments affect adoption and influence their perceptions of new practices. For instance, producers need to adopt specific practices based on their location and its climate, limiting factors, ecology, and existing infrastructure. Producers in areas more susceptible to drought express a higher need for flexibility in practices [46]. Access to social resources such as skilled labor and marketing opportunities are important factors that could prevent producers from adopting a new practice. For instance, if a producer does not have a place to market their crops from an ICLS, it is impractical to implement such a system [47].

## 3. Perceived Practice Characteristics

According to both RAA and DOI, producer perceptions and beliefs surrounding new practices are important factors in the adoption decision [24,25,26]. Producer perceptions of the relative advantage, complexity, compatibility, trialability, observability, and riskiness of a practice should be considered and accounted for in promotion efforts. Perceptions of a new practice or technology are subjective and are informed by the background characteristics previously discussed, prior producer knowledge, and experience, as well as the objective characteristics of the practice or technology.

### 3.1. Relative Advantage

Perceived benefits to the farm and/or environment, or the relative advantage, are often strong predictors of adoption [20,29,48,49]. As one might expect, the perceived benefits of practices are higher among those who adopt practices than those who do not [47]. However, there is a common tendency to discount the benefits of new practices and exaggerate the benefits of the status quo [50]. When applied to the agricultural decision context, this means that the perceptions of new practices are biased toward an overestimation of the potential risks and an underestimation of the potential benefits relative to the current practices [51]. As a result, a marginally beneficial practice may not be perceived as such by a producer, and additional effort may be required to quantify the benefits of a new practice relative to those currently in use. Greater relative advantage not only promotes the overall adoption of practices but can also entice a farmer to learn more about a potential practice [51]. Differential learning incentives lead to greater knowledge about practices perceived to have high benefit–cost ratios relative to practices perceived to have low benefit–cost ratios [51].

### 3.2. Complexity

The more complex a practice is perceived to be, the less likely it will be adopted [52]. Complexity has also been shown to slow the rate of adoption within diffusion of innovation models [36]. Approaches to sustainably intensify grazing land management such as rotational grazing [53,54,55], adaptive grazing management (e.g., multipaddock grazing) [56], or silvopasture entail significantly more complex practices than continuous grazing practices [2]. Other strategies involve ICLS with grazing and pastures with intensively managed diverse cropping systems [15,57,58,59]. Changing grazing practices at the farm level can mean extensive planning, a complete shift in culture and daily routines, and whole system changes, thereby increasing the overall perceived complexity and reducing the appeal of the practice [52]. Researchers have argued that implementing changes at a farm level is a complex and nonlinear process [60]. Therefore, practices that are less complex to implement and can be communicated to producers in a way that reduces their perceived complexity may encourage greater adoption. More complex innovations such as genetic technologies may be more difficult for cattle producers to understand. In these cases, a two-way path rather than a unidirectional diffusion process may be more effective, and intermediaries such as Extension professionals may be needed to encourage adoption [19].

### 3.3. Compatibility

Not all practices are relevant to every operation. The perception of a practice as relevant to the producer’s operation and compatible with current practices is crucial if the goal is to encourage adoption. Moreover, it is important for producers to see practices as easily implemented into their operations as it currently exists. This relevance is paramount. The highest percentage of Louisiana cattle producers surveyed did not adopt new practices because they simply viewed them as irrelevant to their operations [21]. Perceived compatibility will likely change over time, and the adoption of one practice will influence how new innovations are perceived in the future. Arbuckle and Roesch-McNally [29] found that those farming more diverse systems with a greater number of crop types were more likely to perceive new conservation practices as compatible with their existing ones. For those utilizing ICLS, cover crops were compatible with the diverse cropping structure and provided the benefit of additional forage [29]. Producers with a greater number of limiting factors may be more likely to see the value or compatibility of different practices. In King’s 2016 study [46], producers who dealt with drought often saw flexibility in practices as essential. One producer said, “I’ve always tried to keep an open mind and adapt to change, especially when dealing with drought, if you’re not flexible, if you don’t adapt, you don’t survive”.

### 3.4. Trialability

The ability to try out a new practice may encourage producers to consider a new practice. Many producers adopt a practice on a small portion of the land that they operate in order to test out a practice before fully committing. This testing by farmers allows them to gain additional information about a practice and determine how well the results align with their economic, production, and environmental goals [51]. This practice is often used in commercial agriculture through the distribution of trial products to entice producers to purchase said products at a large scale.

### 3.5. Observability

In previous research with cattle producers in Oklahoma and Kansas, visual observations and past experiences were commonly used in day-to-day decision making as well as contributed to how new practices were perceived [44]. The producers interviewed were highly reliant on experience, which was often multigenerational, and observation to guide their decision making. Observability is especially important for practices that are hard to trial due to large start-up costs and extended periods of time before results can be assessed [51]. The ability to observe the success or failure of early adopters will likely influence producer perceptions of the relative advantage of a practice or technology over their current management practices. Given the influence that observability can have on the adoption decisions of other producers, investment in the success of early adopters of a practice or technology can further adoption promotion efforts [61].

### 3.6. Riskiness

Although agricultural science tends to focus on the optimization of production systems in terms of yield per acre, profitability, or some other factor, producers do not necessarily share the same goal, especially if that maximization increases risk or uncertainty. Producers quite rationally work to limit the risk of catastrophic loss outcomes rather than maximizing output or profit from any one aspect of their operation. Evaluating the potential risk involved in the adoption of new practices is essential, especially those practices that have the potential to negatively affect yield [62]. The riskier that a practice is perceived to be by a producer, the less likely it is to be adopted [29]. Practices that are perceived as reducing risk relative to the current practice are readily adopted [36].

## 4. Beliefs

### 4.1. Consequences

Attitudes and beliefs toward practices are also major contributors to the decision-making process [63,64,65,66]. Non-monetary motives, such as maintaining one’s existing quality of life and traditions, often drive decisions regardless of expected profit-maximization pathways [47]. For instance, while it may make economic sense to participate in government-sponsored cost-share programs, many producers are hesitant to “participate in conservation programs because they are uncomfortable with the idea of government control over their land use decisions” [65].

### 4.2. Norms

Farmer choices are made within social contexts of cultural norms and institutions that inform decision making. These norms and the social pressure that they create to adopt an innovation or maintain the status quo are significant. Existing practices are often deeply culturally embedded, and normative practice will influence the perceived relative advantages or disadvantages of new innovations [47]. Perceived social pressure was the primary determinant in the adoption of improved natural grasslands among beef producers in the Brazilian Pampa [67]. The cumulative effects of choices made by individual farmers over time may shift the norms, customs, and institutions that constrain the available choices [68].

Normative beliefs about agricultural practice will be informed by interaction with producers’ social networks and observed practices. The use of other producers as information sources or advisors is common amongst agriculturists [44,69,70]. This can positively or negatively influence sustainable practice adoption, depending upon the practices utilized by social contacts. If producers seek information from others utilizing the same practices, the status quo remains. In contrast, if farmers seek information from those who have already adopted more sustainable practices or come to believe that a practice is widely used, this can encourage adoption [71]. Leveraging a few key producers, or opinion leaders, in a given geographic area could influence many [24]. Shifting social norms to that of conservation can increase the adoption of BMPs [72].

### 4.3. Possibility

Control beliefs are beliefs about the ability to change a behavior or adopt a practice. These include beliefs about the barriers or lack of barriers to adopting a practice, such as sufficient knowledge, sufficient skills, and the availability of qualified technical assistance [73]. Producer beliefs about their financial and physical ability to adopt a practice, as well as the availability of qualified labor, marketing options, infrastructure, and the regulatory environment [47], also fall into this category. Access to the necessary capital may be a better indicator of whether or not a practice will be adopted than the cost itself, given the producer believes the practice to be applicable and feasible. Gillespie and colleagues [21] found that a low number of producers chose not to adopt practices due to cost. However, other studies identified access to capital, which varies substantially among producers, as the best financial predictor of new practice adoption [74]. A 2016 survey of beef cattle producers in the Southern Great Plains indicated that three-quarters of respondents believed that they had some access to financial resources that could be utilized for the adoption of new practices, but the extent of these resources varied widely. One-third of respondents indicated that investment in the adoption of new practices would be contingent upon the prospects of earning an equivalent return, and 17% indicated that they could only invest if a form of cost share was available. However, nearly a quarter (23%) of producers indicated that they could not afford to implement adaptation practices [33]. Insufficient time, labor, and equipment were described as additional constraints to the adoption of new practices. Nearly one-half (43%) of producers indicated that they lacked time, 53% indicated that they lacked labor, and 43% indicated that they lacked the equipment necessary to adopt new practices.

The influences of practice adoption costs and access to capital are further complicated by the fact that the majority of farm households earn some income through off-farm employment. Half of U.S. farms have less than USD 10,000 a year in annual farm sales and rely mostly on off-farm occupations for income [75]. Young producers, i.e., those under the age of 35, are more likely to work off-farm than their older counterparts [76], further compounding time and labor availability constraints.

## 5. Conclusions and Recommendations for Practice and Research

As innovative practices and technologies are developed in the realm of sustainable intensification in animal agriculture, researchers should consider both why producers have or have not adopted existing practices and how the issues discussed here may influence the adoption of new ones. Further research is needed to develop practices that would be more sustainable than those currently practiced if adopted. However, greater attention is needed on the development of practices that are more likely to be adopted or ways to enhance the adoption likelihood of practices and technologies. “Best practice” that is not actually practiced by producers will have little impact on the sustainability of beef cattle production.

Once we move beyond the information deficit model, it is clear that simply sending out a fact sheet detailing the advantages of a practice or increasing a producers’ knowledge on a practice will not necessarily increase the likelihood that it is adopted. Farm and farmer characteristics, perceived practice characteristics, beliefs about the results, social norms, and their own efficacy are all relevant factors in an adoption decision.

By considering the background characteristics of farmers and their farms, new practices and technologies can be more closely aligned with a producer’s actual situation and goals. Although it can be tempting to suggest a “one size fits all” type strategy, “panaceas” to complex socioecological challenges often fail [77]; it is important to communicate with producers to understand them and their perceptions of their operations and current practices and how they may perceive potential practices to increase the overall sustainability of beef cattle production in the future. A “portfolio approach” including a variety of sustainable practices that provides flexibility to ranchers is more likely to include an option that they perceive as beneficial, socially acceptable, and feasible [43].

The relative importance of any particular perceived characteristic will vary from practice to practice as well as between individuals considering their adoption. Reimer et al. [28] identified relative advantage, compatibility, and observability as most salient to conservation practice adoption among Indiana farmers. Perceived practice characteristics also have important interactions that may need to be considered. Trialability and observability can influence perceptions of the riskiness of an innovation. Chibnik [68] distinguished between risk (relatively known probability of success or failure) and uncertainty (unknown probabilities) and asserted the value of trialability and observability to make adoption more risk-like and less uncertainty-like [68]. Activities that embody trialability and observability include adopting a new practice on a small amount of land or seeking more information about the practices and the results that others have had with the practice either within their social group or, more broadly, through social media, asking a crop consultant or other advisor, or identifying state and federally sponsored research. Arbuckle and Roesch-McNally [29] encouraged BMP promoters to focus not just on information about relative advantage but also on quantifying risks and how to manage them (i.e., reducing uncertainty).

Some have suggested that the greatest impact can be achieved by promoting the adoption of conservation practices by the largest farmers since they are both more likely to adopt and control more land [29]. However, we suggest that there may be value in targeting the lower-hanging fruit of the least sustainable practices for behavior change. Although *best* management practices are optimal and researchers’ first choice for producers to adopt, they will have little impact if they are rarely adopted. Perhaps it is important to consider the encouragement of *better* management practices that will be readily adopted by a majority of cattle producers. More progress may be made by shifting the least sustainable practices within a majority of operations to something significantly more sustainable, rather than incrementally improving the practices of producers who are already among those using the most sustainable practices while those utilizing the least sustainable practices continue unchanged. Offering producers more options of potential best (or better) management practices increases the likelihood that they will perceive at least some as compatible and advantageous.

Pigeonholing producers into two groups of adopters and nonadopters is neither helpful nor accurate. There is a continuum of practices utilized by producers that vary in both how sustainable and how intensive they are. By considering factors that influence behavior change in the development of new practices and technologies, researchers can increase the likelihood of developing ones that are more likely to be adopted. Practices that are readily adopted will have a much greater impact on the increased sustainability of beef cattle production than those that may have more theoretical potential but are not adopted by producers. Once practices are ready for implementation, producers need to be provided with the information that they want and need to make an informed choice, not just what we as researchers think that they need to know to implement them [44]. The communication and promotion of new practices and technologies will be more effective if those developing and promoting innovations consider the context, including both the farm and personal characteristics of those whom they want to encourage to adopt them. They should further consider how perceptions of the relative advantage over the current practice, compatibility, perceived complexity, trialability, observability, and riskiness will increase or decrease the likelihood of adoption and what can be done to mitigate negative perceptions. These perceptions will influence and be influenced by livestock producer beliefs about the consequences of the practice, social norms around its adoption or rejection, and their ability to adopt it. Utilizing this conceptual framework as a means of thinking through the factors that will influence the adoption of any practice during the development of behavioral and technical innovations will enhance the likelihood of adoption and increase the success of sustainable intensification efforts.
